# New microsatellite markers for the shallow coral *Madracis auretenra* from the Caribbean

**DOI:** 10.1371/journal.pone.0274895

**Published:** 2022-09-28

**Authors:** Diana Carolina Ballesteros-Contreras, Lina Maria Barrios, Richard Preziosi

**Affiliations:** 1 Faculty of Science and Engineering, Manchester Metropolitan University-MMU, Manchester, United Kingdom; 2 Instituto de Investigaciones Marinas y Costeras de Colombia-INVEMAR, Santa Marta D.T.C.H., Colombia; 3 School of Biological and Marine Sciences, University of Plymouth, Plymouth, United Kingdom; National Bureau of Plant Genetic Resources, INDIA

## Abstract

Coral reefs species represent one-third of all marine species described in the ocean. They are also responsible for providing habitat and support to different species. Recently, Caribbean coral reefs ecosystems have suffered an alarming decrease in their populations as a result of overexploitation. *Madracis auretenra* in particular, is a widespread shallow coral in marine protected areas (MPAs) of the Caribbean. Due to the important role of MPAs as a biodiversity conservation tool, this species can be used as a model to estimate its dispersion/migration among Caribbean reefs through the use of informative genetic markers (microsatellites) specifically designed for it. Seventeen new polymorphic microsatellites markers for *M*. *auretenra* were developed and tested in 330 samples from Colombia, Guatemala, Curacao and Barbados. The gene diversity (1-D) ranged from 0.482 to 0.903, while the evenness ranged from 0.456 to 0.884 after clone removal. The expected heterozygosity (He) ranged from 0.347 to 0.742, while the observed heterozygosity (Ho) ranged from 0.078 to 0.578. The specificity of our microsatellites shows the potential use of these markers in *a-posteriori* analysis to detect population structure at different spatial scales, where *M*. *auretenra* is reported.

## Introduction

Scleractinian corals are a key species in tropical reef ecosystems, allowing different organisms to use their three-dimensional structures as refuge and as nurseries for some fishes of economic importance [1]. As a result, coral reefs are one of the most diverse ecosystems in the world, providing protection to the coast, and other ecosystems from erosion. Reefs are also considered as key in the development of coastal zones, producing a considerable income to the tourism industry [2]. Coral species also represent one-third of all marine species described in the ocean [3]. However, 60% of the coral reefs around the world are in danger due to natural and anthropogenic factors such as sedimentation from rivers, marine contamination, and overfishing [4, 5]. The Caribbean Sea is considered as a vulnerable area due to the decline in live coral coverage, which reached 40% during the last decade; likewise, the coral recovery after disturbances in the Caribbean seems to be slower compared with Indo-Pacific reefs due to the limitation in the replacement of larvae from neighbouring reefs [6]. However some corals, such as those from the genus *Madracis*, are found in diverse environments, including tropical areas, temperate waters, shallow and deep habitats [7]. The genus *Madracis* also shows a high phenotypic plasticity (variable morphotypes) that accommodates to the variety of habitats, environmental conditions or hybridization processes, generating sometimes difficulties in their identification [8, 9]. These morphological features promoted its phylogenetic position relocation, from the Pocilloporidae to the Astrocoeniidae family, but several studies based on different nuclear and mitochondrial makers show that *Madracis* is closely related to *Pocillopora* [7].

The zooxanthellae coral *M*. *auretenra* has an abundant distribution across the Caribbean, giving it the position of “Least Concern” under the parameters of IUCN (International Union for Conservation of Nature and Natural Resources), representing high richness values of associated species in the area [10]. *Madracis auretenra* develops an optimal growth between 1 and 20 m depth, showing patches that can reach more than 5m in diameter [11]. This is a hermaphroditic coral, with male and female gametes in fertile polyps, similar to other species of *Madracis* in the Caribbean, but with small differences in reproductive strategies, cycles and fecundity [12]. Given the vulnerability of coral reefs in the Caribbean, some marine reserves have been created to increases the reproductive capacity and transport of larvae among different habitats [13]. However, for the design and management of the marine reserves, molecular markers such as microsatellites are commonly used due to the high variance and robust information they provide [14]. Due to the presence of *M*. *auretenra* in different localities throughout the Caribbean, the aim of this study was to develop informative microsatellite markers for the species *M*. *auretenra*, which can be used in future ecological studies to understand the connectivity among shallow reefs in the Caribbean Sea.

## Materials and methods

### Study area

The sampling was carried out in 17 localities in Colombia (174 fragments), four in Curacao (80 fragments), three in Barbados (78 fragments) and one locality in Guatemala (23 fragments) (S1 Table). All the samples were collected through scuba diving, taking into account to keep at least 5 meters of distance between colonies at depths of 5m to 25m. The samples were kept in alcohol 96% or DMSO and then, stored at -20° C. The samples from Colombia were collected under the permits obtain by the Institute for Marine and Coastal Research-INVEMAR; Curacao, under the permits obtain by Carmabi Fundation; Barbados, under the permits obtain by the Coastal Zone–Management Unit from the Ministry of Maritime Affairs and the Blue Economy; and Guatemala under the permits obtain by the National Council of Protected Areas-CONAP (Consejo Nacional de Areas Protegidas). The samples were exported to Manchester Metropolitan University-MMU under the CITES permits 41449 (25th January 2017) and 43908 (6th May 2019) obtained by the Colombian Ministry of Environment and Sustainable Development (Ministerio de Ambiente y Desarrollo Sostenible). The samples from Curacao were exported under the Institute’s CITES agreement between Carmabi Fundation (Curacao), with the collaboration of Dr. Mark Vermeij, and Natural History Museum of London (UK), with the collaboration of Dr. Nadia Santodomingo, on 29^th^ of June 2018. The samples from Barbados and Guatemala were exported to MMU under the CITES permits 04210 (27^th^ of March 2019) obtained by the Barbados Ministry of Environment and Drainage and 000605 (14^th^ April 2019) obtained by the National Council of Protected Areas-CONAP (Consejo Nacional de Areas Protegidas), respectively.

### DNA extraction, microsatellites development and genotyping

To standardize the DNA extraction protocol, fragments of *M*. *auretenra* donated by the Blue Planet Aquarium (Birmingham, UK) in 2015 and kept alive in aquariums at MMU were used. The DNA tissue and blood extraction kit from Qiagen were used, and the DNA concentration and quality were measured using a NanoDrop (Thermo Scientific). Twenty-five samples were eliminated due to the low quality and quantity of DNA, leaving 330 samples for the microsatellite development. Two DNA samples of *M*. *auretenra* from the MMU’s aquarium (good quality and quantity of DNA) were chosen for sequencing and normalized to 50ng using the Nextera® DNA sample Preparation Kit. The samples were sent to the Illumina MiSeq sequencing facility at the University of Manchester—UoM (UK) to perform a paired sequencing (2x250bp). The sequencing data was analysed on the Galaxy Centaurus Server platform (https://palfinder.ls.manchester.ac.uk), using the bioinformatics tool Palfinder to identify sequences containing repeat motifs and optimised the microsatellite development [14]. From the process, 61 potentially amplifiable loci (PALs) were obtained and 36 PALs (21 tri- and 15 tetra-nucleotide motifs) with satisfactory quality assessment by FastQC reports were chosen [15].

For the primers designed the Type-it Microsatellite PCR QIAGEN kit parameters was used (melting temperature, annealing temperature and primer length). Two DNA samples per location (50 in total), were used to explore the amplification of the 36 PALs–microsatellites primers in 10 μL reaction mixes containing: 5 μL of Master Mix (Type-it Microsatellite PCR QIAGEN Kit), 3 μL H2O molecular grade, 1μl Primer mix and 1μl DNA (20 ng/μl). The PCRs were run in a TECHNE thermocycler under the following conditions: (1) denaturation 95°C/5 min; (2) 32 cycles including 95°C/30 sec for denaturation, 60°C/1.5 min for annealing, and 72°C/30 sec for elongation; (3) a final extension at 60°C/30 min; (4) an endless holding at 4°C.

The PCR products were electrophoresed in a 0.8% agarose gel to confirm amplification of the microsatellites. After the exploration, 12 microsatellites primers were excluded as a result of irregular amplification in the samples. The 24 remain microsatellites primers were redesigned adding the universal tail sequence Blackett A: GCCTCCCTCGCGCCA [16] and M13-mod B: CACTGCTTAGAGCGATGC [17], which allow distinguishing among amplified fragments by the labelling with fluorescent dyes (6-FAM and ROX, accordingly). The redesigned 24 microsatellites primers were tested in the remained 280 samples (good DNA samples: 330, previous amplification: 50 samples) using the program Multiplex_Manager [18] to perform multiplexes. Each amplification run used positive and negative controls using 8 samples from the 50 samples previously tested. The new reaction mixes contained 5 μL of Master Mix (Type-it Microsatellite PCR QIAGEN Kit), 3 μL H2O molecular grade, 1μl Primer mix (Pre_laballed Forward + Reverse + Fluorescence 6-FAM or ROX + H20 molecular grade) and 1μl DNA (20 ng/μl). The PCR conditions and the confirmation were the same as described above. In addition, a primer’s specificity test was performed using samples of *M*. *myriaster*, *Montipora* sp. and *Antillogorgia* sp., under the same amplification conditions. None of the primers amplified on these samples, confirming their specificity. The genotyping was performed in the 330 samples. The fluorescence labelled PCR products were sent to the University of Manchester DNA Sequencing Facility (UK) and to the Core Genomics Facility at the University of Sheffield (UK), in a mix of: 9μl of HiDi Formamide, 0,2μl of the Liz 500 (GeneScan™ 500 LIZ®) and 0,8μl of the PCR product (including the positive and negative controls). The products were sized in both places using the capillary electrophoresis Applied Biosystems 3730 DNA Analyser (enabling size discrimination within the range of 20 to 600 base pairs using a range of dyes).

### Data analyses

The software R was used to analyse the PCR products, performing data analyses with the package “Fragman” [19] to read FASTA files with different fragments sizes for each loci, in each sample obtained after the genotyping. For each allele sizes, the values were adjusted with the positive control. The “MsatAllele” package [20] was employed to bin the fragments sizes (Fragman output), assigning to a set of defined alleles the closest size for each marker. Later, the true allele set was determined using the known repeat length and using the histograms of observed fragment lengths, before and after the binning. The package “Poppr” [21] was used to assess the frequency of missing alleles across primers and localities. In addition, the number of clones present in the sampling was investigated by counting multi-locus genotypes throughout localities in “Poppr”, and only one genotype per clone were kept for the analyses.

The allelic diversity was exanimated at each locus before and after the removal, using Simpson’s Index, in order to identified changes with the elimination of the clonal samples using “Poppr” [21]. The expected heterozygosity, gen diversity and evenness for each locus was evaluated using Nei’s index. The package “FreeNa” [22] was used to calculate the null allele frequency in the loci, using the expectation maximization (EM) algorithm in 1000 bootstrap resamples in each level (Country, Department, and Locality). The function “hw.test” of the package “pegas” in R [21] was employed to determinate the Hardy-Weinberg Equilibrium (HWE) in the localities, using the χ2-test, based on the expected genotype frequencies calculated from the allelic frequencies, and an exact test based on Monte Carlo permutations (1000) of alleles [23]. The obtained values were corrected by BH (Benjamini-Hochberg) method in R. The linkage disequilibrium p-values were calculated using “Genepop” [24, 25] in R. This last analysis in R was performed by locality for all pairs of loci, with a Bonferroni correction.

## Results

Twenty-four microsatellite loci were analysed in Caribbean samples of *M*. *auretenra*. Due to the poor binning performance, four loci (3, 5, 13 and 28) were removed. The remaining 20 microsatellite loci were analysed in “Poppr”, using a criterion of 20% of missing data across all samples; from this process the loci 4,10 and 27 were excluded. The final 17 microsatellites were tested in the selected 330 samples of *M*. *auretenra*. Seven samples from the 330 were putative clones in the localities of Cabo Tiburon (1 sample), Socorro (2 samples), Varadero (3 samples) and Folkstone (1 sample); the copies of this identical multi-locus genotypes were eliminated. From the remaining 323 samples, the localities with less than 10 samples were excluded (Cinto, Neguanje, Bajo Carey and Tesoro Oriental). Also, the sampling for the islands COCA and ARENA was grouped as Islas del Rosario; In a similar way, the sampling for SOC, MAR and VEN were grouped as Isla Fuerte; the decision was taken in order to include this sampling in the analysis, increasing the number of sampling per locality and the short distances between patches sampled (< 2.5km^2^) (S1 Table). A total of 313 samples were used in the data analysis. The allelic diversity at each locus was not affected after clone removal in Poppr. The gene diversity (1-D) ranged from 0.482 to 0.903, while the Evenness ranged from 0.456 to 0.884 after clone removal. The expected heterozygosity (He) using Nei’s (1978) ranged from 0.347 to 0.742, while the observed heterozygosity (Ho) ranged from 0.078 to 0.578 (Table 1).

**Table 1:** Allelic diversity of *M*. *auretenra* microsatellite loci. Gene diversity measure (1-D), Evenness, observed (Ho) and expected (He) heterozygosity.

Locus	1-D	Evenness	Ho	He
**Maur_1**	0.609	0.456	0.259	0.402
**Maur_6**	0.839	0.752	0.326	0.7
**Maur_7**	0.622	0.645	0.078	0.348
**Maur_8**	0.702	0.678	0.578	0.56
**Maur_12**	0.745	0.690	0.166	0.617
**Maur_14**	0.858	0.614	0.518	0.718
**Maur_16**	0.761	0.783	0.108	0.462
**Maur_17**	0.872	0.798	0.103	0.582
**Maur_20**	0.889	0.846	0.376	0.742
**Maur_21**	0.903	0.862	0.44	0.733
**Maur_23**	0.663	0.534	0.328	0.558
**Maur_24**	0.780	0.744	0.517	0.666
**Maur_26**	0.482	0.709	0.078	0.347
**Maur_30**	0.860	0.884	0.154	0.473
**Maur_34**	0.816	0.878	0.115	0.464
**Maur_35**	0.889	0.755	0.153	0.606
**Maur_36**	0.743	0.671	0.27	0.626

**Fig 1:**
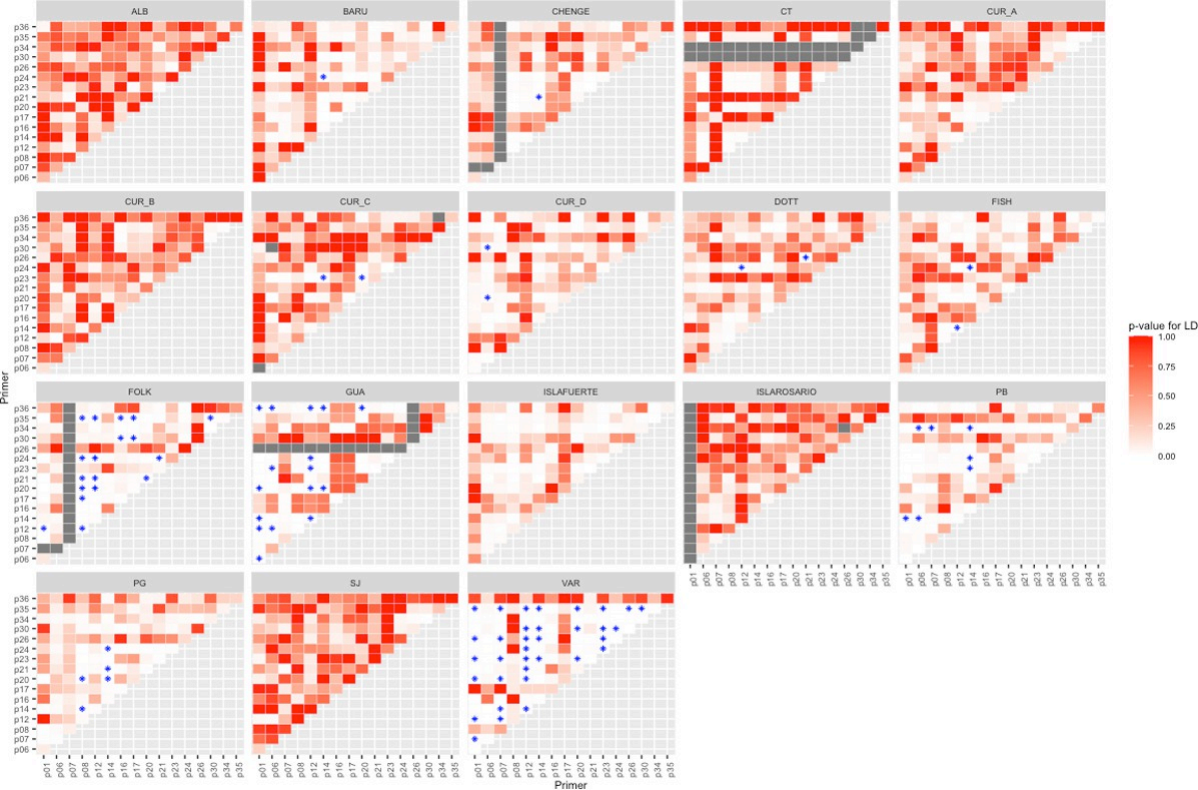
Linkage disequilibrium per locality. Blue stars show the p- values <0.05 after the Bonferroni correction. Clear tones (white) are the p-values < 0.05 without the Bonferroni correction. Grey colours show the missing or not enough data for the loci (<than four samples per locality).

The primer sequences were described by motif, universal tail used in the multiplex, size range, number of alleles observed per locus (between 4 and 18) and GenBank accession number of the 17 microsatellite loci in Table 2. After testing the frequency of null alleles in FreeNa and taking into account that the ranges [22] are usually from 0.0 to 0.2 (low < 0,05; medium < 0,20; high > 0,2), high values were found (> 0.25) for some markers. After the analysis, no differences were found between uncorrected and corrected F_ST_ at locality or department scales (Table 3). Some of the loci and population showed a significant deviation from Hardy Weinberg Equilibrium (p<0.001) (Table 4). Using Genepop, the Bonferroni correction was applied on the linkage disequilibrium p-values (LD). Values of LD between pairs of loci were found in 11 localities of the Caribbean: five localities in Colombia (BARU, CHENGUE, PG, PB and VAR), two in Curacao (Sites C and D), and all the localities in Barbados (FOLK, FISH, DOTT) and Guatemala (Izabal). The higher values were in the locality of FOLK (Barbados), Izabal (Guatemala) and VAR (Colombia) (Fig 1).

**Table 2:** Characteristics of novel microsatellite loci developed for *M*. *auretenra*: Locus name, forward and reverse primer sequences, motif, universal tail (fluorescence label), base pairs size range, number of alleles and GenBank accession number.

Locus	Primer Sequences (5’ - 3’)	Motif	Universal Tail	Size range (bp)	No Alleles	GenBank accession No
**Maur_1**	F: AACGATCAAAGTCGATGGAGACGTAAGGC	AAAC	Blackett A	271–442	13	MT799827
	R: TCCACTCAAGAACAATGTTGATACACAGGC					
**Maur_6**	F: CGACTGGATTGAAGTTACTAGAGTGCGG	TCCG	Blackett A	208–460	12	MT799828
	R: CTCAAGTCATCCAAGGTCAGCAGCG					
**Maur_7**	F: AATAGGCGCGTTCAGAGAACAAGGG	AATC	Blackett A	297–406	10	MT799829
	R: CATGTCTAATCCCCATGATTGCGG					
**Maur_8**	F: TTCTTATTCTGTGCTTCCTTTGCTGGG	TCTG	Blackett A	240–290	11	MT799830
	R: ATGCCAGGTTGCCTCTGATTGGC					
**Maur_12**	F: GTGAATAAATGAATAACTGAGTACCTCCCG	AAAT	Blackett A	306–476	12	MT832345
	R: GAAGAAACGTCAACAGTGAGAGGGG					
**Maur_14**	F: TTTAGGAAGGGAAGTGCCTGTTCCG	AAAT	Blackett A	252–346	18	MT832346
	R: TCACGGCTAATAAATTTCACGTGCG					
**Maur_16**	F: CTTCGGCAGGATCATTTGTAATCGG	AAC	Blackett A	250–281 (410)	10	MT832347
	R: TTTAGCAGCTTGGTGCCAAACCC					
**Maur_17**	F: GAGTTCCAAGGAGTTTTGAATTGCCC	AGT	Blackett A	257–314	15	MT832348
	R: GATGTAGAATCGCAGGTTCCTTGGG					
**Maur_20**	F: GGATAATGTTGTAGTCCATGCCTTGCC	ATT	M13-mod B	270–357 (476)	16	MT832349
	R: GCAATTAATTATCCCATTGAAGCTCTGGC					
**Maur_21**	F: AAGTAGTTGCTTGACTCTTTTCTGAAGCG	TTC	M13-mod B	165–223	16	MT832350
	R: ACCTCCACCTTGACAGCTCTTTCCC					
**Maur_23**	F: TATGCGCACATTCAACTAGCATCCC	ATT	M13-mod B	281–434	10	MT832351
	R: TGTTAAGGCTTGGTTTCTTCATGCG					
**Maur_24**	F: GAAATCTTTGTTTCAAGGAGGAAGGGG	TTC	M13-mod B	230–328	13	MT832352
	R: TCTACCTTGGCTTGTTCATCAGGGG					
**Maur_26**	F: GTGGATGAGGAAGAGAGCAGTGGC	ATC	M13-mod B	325–337	4	MT832353
	R: CTGTCATGAGATCCAAACCTCCGC					
**Maur_30**	F: TCCATAGTCTCACGATTGAGCG	ATC	M13-mod B	304–336 (425)	11	MT832354
	R: GAACAGTATGTTTACCCAGATCCC					
**Maur_34**	F: CCTGGCTCCAAGTTGAAAGTAGGC	ATC	M13-mod B	310–441	9	MT832355
	R: TCAAGTTTGAAGACTGCAAGTAATCCC					
**Maur_35**	F: GGGTATACACCCTGAAGTTTCACATAGCC	ACC	M13-mod B	255–355	18	MT832356
	R: CTCCGTAGGAATCCGAGCATTACCC					
**Maur_36**	F: TCATGGTTGAGAGGTTCATATTTTAGCCC	ATT	M13-mod B	250–313 (325)	15	MT832357
	R: ATAAAACACAAAGGCTGGTCACGGC					

**Table 3:** Null alleles frequency (NAF) and global F_ST_ (G F_ST_), calculated with and without ENA correction, for null alleles in different scales (Country, Department and Locality).

Locus	NAF	G F_ST_ Country	G F_ST_ ENA Country	G F_ST_ Department	G F_ST_ ENA Department	G F_ST_ Locality	G F_ST_ ENA Locality
**Maur_1**	0.197	0.193	**0.183**	0.217	**0.210**	0.266	**0.259**
**Maur_6**	0.288	0.089	**0.064**	0.119	**0.094**	0.198	**0.162**
**Maur_7**	0.344	0.503	**0.428**	0.524	**0.453**	0.503	**0.438**
**Maur_8**	0.071	0.196	**0.188**	0.190	**0.184**	0.209	**0.206**
**Maur_12**	0.323	0.064	**0.054**	0.129	**0.102**	0.173	**0.139**
**Maur_14**	0.195	0.134	**0.138**	0.139	**0.139**	0.203	**0.186**
**Maur_16**	0.369	0.428	**0.374**	0.440	**0.375**	0.428	**0.358**
**Maur_17**	0.408	0.318	**0.292**	0.330	**0.292**	0.378	**0.331**
**Maur_20**	0.264	0.123	**0.092**	0.143	**0.118**	0.169	**0.137**
**Maur_21**	0.245	0.122	**0.092**	0.131	**0.102**	0.203	**0.162**
**Maur_23**	0.218	0.133	**0.112**	0.189	**0.158**	0.217	**0.189**
**Maur_24**	0.153	0.100	**0.081**	0.120	**0.100**	0.154	**0.125**
**Maur_26**	0.285	0.115	**0.132**	0.225	**0.216**	0.299	**0.263**
**Maur_30**	0.383	0.394	**0.349**	0.462	**0.427**	0.484	**0.445**
**Maur_34**	0.384	0.361	**0.358**	0.407	**0.390**	0.449	**0.405**
**Maur_35**	0.384	0.204	**0.164**	0.233	**0.188**	0.335	**0.278**
**Maur_36**	0.280	0.108	**0.103**	0.148	**0.145**	0.197	**0.181**

**Table 4:** P- values to test deviation from Hardy Weinberg Equilibrium for each population and locus. Significant (p<0.001) values are presented in bold.

Locality	1	6	7	8	12	14	16	17	20	21	23	24	26	30	34	35	36
ALB	0.02	**0.00**	**0.00**	0.14	0.01	**0.00**	0.00	**0.00**	**0.00**	0.01	0.78	0.01	0.11	0.43	**0.00**	**0.00**	**0.00**
CT	1.00	**0.00**	0.00	0.07	**0.00**	**0.00**	0.00	**0.00**	0.11	0.01	0.01	**0.00**	0.92	1.00	1.00	0.00	0.03
ISLAFUERTE	0.00	0.02	0.03	0.01	0.02	0.01	0.00	0.00	0.23	**0.00**	0.11	0.00	0.00	0.00	**0.00**	0.01	**0.00**
ISLAROSARIO	1.00	0.04	**0.00**	0.95	**0.00**	0.00	0.00	0.00	**0.00**	0.00	0.01	0.83	0.00	0.01	0.00	**0.00**	0.00
BARU	0.92	**0.00**	0.00	0.09	0.01	**0.00**	0.00	**0.00**	0.09	**0.00**	0.02	0.00	0.00	**0.00**	0.00	0.00	**0.00**
PB	0.00	**0.00**	0.02	1.00	**0.00**	**0.00**	**0.00**	**0.00**	0.00	**0.00**	0.01	0.42	**0.00**	**0.00**	**0.00**	0.00	**0.00**
PG	0.00	**0.00**	**0.00**	**0.00**	**0.00**	**0.00**	**0.00**	**0.00**	**0.00**	**0.00**	0.00	0.00	0.09	**0.00**	0.00	**0.00**	0.00
SrJUAN	0.05	**0.00**	0.05	0.99	**0.00**	0.00	**0.00**	**0.00**	0.00	0.00	0.08	0.04	0.03	0.00	**0.00**	0.00	0.01
VAR	**0.00**	0.04	**0.00**	**0.00**	**0.00**	**0.00**	**0.00**	**0.00**	0.00	**0.00**	**0.00**	**0.00**	**0.00**	**0.00**	**0.00**	**0.00**	**0.00**
CHENGE	**0.00**	0.01	1.00	**0.00**	0.14	**0.00**	0.12	0.01	0.01	0.21	0.01	0.10	0.31	0.41	0.11	0.00	0.06
CUR_A	0.96	0.01	**0.00**	0.97	**0.00**	0.99	0.00	**0.00**	**0.00**	0.03	0.00	0.01	0.00	0.00	0.00	0.01	**0.00**
CUR_B	**0.00**	0.13	**0.00**	1.00	**0.00**	0.92	**0.00**	0.02	0.08	0.01	**0.00**	0.05	**0.00**	**0.00**	**0.00**	**0.00**	**0.00**
CUR_C	**0.00**	**0.00**	**0.00**	**0.00**	**0.00**	0.64	**0.00**	**0.00**	**0.00**	**0.00**	0.39	0.00	**0.00**	**0.00**	**0.00**	**0.00**	**0.00**
CUR_D	0.87	**0.00**	**0.00**	0.12	**0.00**	**0.00**	**0.00**	**0.00**	**0.00**	0.00	**0.00**	**0.00**	**0.00**	**0.00**	0.01	**0.00**	**0.00**
DOTT	**0.00**	**0.00**	0.00	**0.00**	0.00	**0.00**	0.08	**0.00**	0.17	**0.00**	0.00	**0.00**	0.68	**0.00**	**0.00**	**0.00**	**0.00**
FISH	**0.00**	**0.00**	**0.00**	**0.00**	**0.00**	**0.00**	**0.00**	**0.00**	**0.00**	0.02	**0.00**	0.24	**0.00**	**0.00**	**0.00**	**0.00**	**0.00**
FOLK	**0.00**	**0.00**	1.00	0.03	**0.00**	**0.00**	**0.00**	**0.00**	**0.00**	0.05	0.53	0.00	0.01	**0.00**	**0.00**	**0.00**	**0.00**
GUA	0.00	**0.00**	0.00	**0.00**	**0.00**	**0.00**	0.88	0.88	**0.00**	**0.00**	**0.00**	**0.00**	1.00	0.96	0.88	**0.00**	**0.00**

## Discussion

The amplification of 24 microsatellite loci in 330 samples was obtained, but only 17 markers were chosen. Due to the poor binning and missing data across all samples in seven microsatellite loci, several PCRs were performed using the same fragment in order to discard possible amplification errors as “false homozygote” (random sampling in the DNA template and deficiency of amplification) [26], which is a common response for very low DNA quantity [27]. The seventeen polymorphic microsatellite markers for *M*. *auretenra* were tested in multiplex of two and three-primers due to the similar size range, combining the two different Universal tails to reduce cost in the full analysis. From the 330 samples used to test the microsatellites, seven samples were eliminated due to the identical multi-locus genotypes in the same locality, suggesting that the sampling method (5-meter distance between colonies) showed a good result to reduce the number of clones. Also, ten samples from localities with less than 10 fragments were excluded for the data analysis. *Madracis auretenra* is a coral that displays a remarkable degree of morphological variation along environmental gradients and geographic regions, term known as phenotypic plasticity, which is mainly to its branching morphology and asexual mode of reproduction [9]. Due to the asexual reproduction mode and its morphological variation, the identification efforts and taxonomic practices during the last 40 years, the species has been misidentified several times as *M*. *mirabilis*, a synonym of the deep coral *M*. *myriaster* [28]. In this sense, the characterized microsatellites bring new tools for future taxonomists and ecologist, as a complementary tool to improve traditional taxonomic identification [7, 29].

The data also showed a widely variable number of alleles per locus, with high diversity (1-D), high expected heterozygosity (He) and equality allele distribution (Evenness). All these parameters are considered as a sign of informative loci due to the polymorphic characteristic that can be used for future analysis to detect population structure in different spatial scales. Some markers exhibited high frequency of null alleles, after following the ranges suggested by other authors [22]. Those null alleles represent substitutions and indel mutations in their annealing sites, preferential amplification of short alleles, or slip-pages during the PCR amplifications [22]. Also, the presence of null alleles in corals have been reported previously, when using microsatellites in species such as *Montastrea caveronsa*, *Porites astreoides*, *Galaxea fascicularis* and species of the genus *Millepora* [30–32]. Due to the presence of null alleles in the data, the estimation of the Global F_ST_ values with ENA correction for null alleles was performed, letting the new microsatellites to be used in further analysis and reduce the overestimation in possible differences among populations. The majority of the loci, in most of the localities, deviated significatively from Hardy Weinberg Equilibrium, with p-values <0.001. This deviation could be a consequence of the evidence of inbreeding and null allele presence [33]. Also, the HWE deviation is a common characteristic of the life history for Scleractinian corals and had been reported previously in species such as *Pocillopora damicornis*, *Montastrea cavernosa* and *Porites astreoides* [31, 32]. Furthermore, the results on linkage disequilibrium after Bonferroni correction for multiple comparisons showed significant p-values in eleven of the 18 localities. However, the decision to keep all the loci was made because no relationships between the localities or the loci were found and all the loci can be used on future analysis in population structure, genetic linkage and as a comparison for other species. The specificity of the microsatellites developed for *M*. *auretenra* was confirmed by the null amplification in other corals such as *M*. *myriaster*, *Montipora* sp. and *Antillogorgia* sp., showing the potential use of these markers as a complementary taxonomic identification tool; they can also improve the individual resolution at population level.

In addition, the utility of specific microsatellites have been used in other groups as a useful tool in conservation studies [34] around the word, as the octocoral *Eunicella verrucosa* in England and Wales [35], multiple *Pocillopora* lineages in the north-western Pacific [36] or *Montastrea cavern*osa in the Caribbean [37]. Therefore, the new microsatellites presented here can be used in the future to expand the knowledge about connectivity among shallow coral reefs in the tropic. These markers will also allow the improvement on the design and management of marine reserves as MPAs in the Caribbean and Atlantic regions.

## Supporting information

S1 Table *Madracis auretenra* sampling information for the 18 Caribbean Sea localities. MPA: Marine protected area name. * grouped localities. The number of the samples belong to final set of 313 samples used in the data analysis.
(DOCX)
