# Supplementary material for: New microsatellite markers for the shallow coral Madracis auretenra from the Caribbean
Source: PLoS One. 2022 Sep 28;17(9):e0274895. doi: 10.1371/journal.pone.0274895 (PMC9518922; doi:10.1371/journal.pone.0274895)
Supplement: S1 Table — MPA: Marine protected area name. * grouped localities. The number of the samples belong to final set of 313 samples used in the data analysis. (DOCX) [file pone.0274895.s001.docx]

**S1 Table A**. *Madracis auretenra* sampling information for the 18 Caribbean Sea localities. MPA: Marine protected area name. * grouped localities. The number of the samples belong to final set of 313 samples used in the data analysis.

| Country | Department | Location | ID | Coordinates | Samples used | MPA |
| --- | --- | --- | --- | --- | --- | --- |
| Barbados | Barbados | Folkestone | FOL | 13.190575, 59.6408389 | 24 | Folkestone Marine Reserve |
|  |  | Fisherman | FISH | 13.1830167, 59.6471278 | 28 | Outside |
|  |  | Dottins | DOTT | 13.1791778, 59.6496639 | 25 | Outside |
| Curacao | Curacao | C_Site_A | CUR_A | 12.3754444, 69.158025 | 18 | Outside |
|  |  | C_Site_B | CUR_B | 12.2285167, 69.0927389 | 16 | Outside |
|  |  | C_Site_C | CUR_C | 12.1212139, 68.9696639 | 20 | Outside |
|  |  | C_Site_D | CUR_D | 12.04145, 68.8163806 | 20 | CARMABI Marine Reserve |
| Colombia | Magdalena | Chenge | CHENGE | 11.3255556, 74.1283333 | 13 | Tayrona National Park |
|  | Bolivar | Varadero | VAR | 10.309722, 75.58916 | 21 | Outside |
|  |  | Punta Bota | PB | 10.2872222, 75.5944444 | 16 | Corales del Rosario and San Bernardo National Park |
|  |  | Punta Gigante | PG | 10.2586111, 75.6136111 | 15 | Corales del Rosario and San Bernardo National Park |
|  |  | Sr Juan | SrJUAN | 10.26, 75.6175 | 13 | Corales del Rosario and San Bernardo National Park |
|  |  | Baru | BARU | 10.2552778, 75.62 | 11 | Corales del Rosario and San Bernardo National Park |
|  |  | ***Coca***  ***Arena*** | *ISLAROSARIO** | 10.1680556, 75.62  10.152778, 75.746111 | 10 | Corales del Rosario and San Bernardo National Park |
|  | Córdoba | ***Socorro***  ***Venados***  ***Marijao*** | *ISLAFUERTE** | 9.4058333, 76.200278  9.385, 76.1694444  9.38027778, 76.1708333 | 13 | Corales del Rosario and San Bernardo National Park |
|  | Urabá Chocoano | Cabo Tiburón | CT | 8.67027778, 77.3586111 | 11 | Outside |
|  | SAI | Albuquerque | ALB | 12.13972, 81.86388 | 16 | Seaflower Biosphere Reserve |
| Guatemala | Izabal | Puerto_Barrios | GUA | 15.8887278, 88.1610778 | 23 | Punta Manabique Wild Life Reserve |
